# Is the combination of acupuncture and Western medicine superior to monotherapy in the treatment of patients with Alzheimer’s disease: A protocol for systematic review and network meta-analysis

**DOI:** 10.1097/MD.0000000000032093

**Published:** 2022-12-16

**Authors:** Xinran Wei, Yan Tan, Chao Ke, Yang Cao, Zhengrong Xie, Liumei Yuan, Jiang Pan, Wei Zhang

**Affiliations:** a Department of Acupuncture, Moxibustion, Tuina and Rehabilitation, The First Affiliated Hospital of Hunan University of Traditional Chinese Medicine, Hunan province, China; b Hunan University of Traditional Chinese Medicine, Hunan province, China.

**Keywords:** acupuncture, Alzheimer disease, network meta-analysis, protocol

## Abstract

Alzheimer disease (AD) is a common cause of dementia, and there are still a lack of treatment options to reverse or prevent disease progression. Existing evidence shows that acupuncture has advantages in the treatment of AD, but whether the efficacy of acupuncture belongs to the placebo effect remains controversial, and there is no strict systematic review and network meta-analysis to evaluate the efficacy and safety of acupuncture combined with Western medicine in the treatment of AD.

**Methods:**

From the inception to February 2023, the Embase, Latin American and Caribbean Health Sciences Literature, Medline, the Cochrane Collaboration’s Controlled Clinical Trials, Scopus, China Biomedical Literature Database, Wanfang Database, China National Knowledge Infrastructure, and Australian Medical Index will be searched using the key phrases “acupuncture,” “warm needling,” “electroacupuncture,” “Alzheimer disease,” and “cohort” for all relevant studies. Quality assessment of all studies included in this review will be independently assessed by 2 reviewers using the Cochrane Collaborations tool. When significant heterogeneity is indicated, we will find the source of heterogeneity by subgroup or sensitivity analysis.

**Discussion:**

This study will evaluate the efficacy of acupuncture combined with Western medicine in improving cognitive function and activities of daily living in AD patients. The results of this study will verify whether the efficacy of acupuncture in the treatment of AD belongs to the placebo effect, which will also provide a reference for the clinical use of acupuncture combined with Western medicine in the treatment of AD.

## 1. Introduction

Alzheimer’s disease (AD) is a chronic degenerative disease of the central nervous system characterized by progressive cognitive dysfunction. Approximately 36 million people worldwide are currently affected by the disease, and it is estimated that AD will affect more than 100 million people by 2050, placing a heavy burden on families, societies and economies.^[1,2]^ The prevalence of AD is higher in women than in men and is more common in older patients over the age of 70.^[3]^ The etiology and pathogenesis of AD have not been elucidated. The typical pathological changes of AD are neuroinflammatory plaques, neurogenic fiber tangles, neuronal loss and gliosis.^[4]^

Modern medical treatment of AD is mostly symptomatic with cholinesterase inhibitors such as donepezil and carbalatine. Although drug therapy alone can improve cognitive function and delay the disease to a certain extent, the course of AD is long and there are more adverse events in long-term application. According to its clinical manifestations, it can be classified as “dementia” or “demented disease” in Chinese medicine. The understanding of the etiology and pathogenesis of AD has tended to be unified, with the deficiency of the kidney as the root cause and the phlegm clotting and blood stasis as the symptoms.^[5–7]^ Acupuncture has a long history of 3000 years and is widely used for the prevention and treatment of AD. It can unblock the meridians, raise the heart and Yang, nourish the liver and kidney, calm the mind, and fill the marrow, thus improving the cognitive function and the quality of daily life.^[8,9]^

Based on this, we raised an important clinical question: Among these Chinese and Western medical therapies, whether combined acupuncture and Western medicine treatment is more effective and safer than monotherapy in patients with AD. Unlike traditional pairwise meta-analyses, network meta-analyses can summarize both direct and indirect evidence and evaluate relative efficacy in multiple treatment comparisons. More importantly, it can provide a ranking of treatment options based on their effectiveness. Therefore, we should conduct a systematic review and network meta-analysis to summarize the evidence for various therapies and to identify the most effective AD treatments.

## 2. Materials and methods

The protocol of systematic literature review and meta-analysis was structured to adhere to the PRISMA (Preferred Reporting Items for Systematic Reviews and Meta-Analyses) Guidelines.^[10]^ The systematic review protocol was registered on PROSPERO (CRD42022368796). Ethical approval was not necessary because the present meta-analysis was performed on the basis of previous published studies.

### 2.1. Data sources and search strategy

Search strategies will be created and applied to the following electronic databases: Embase (Elsevier, 1980–2022), Latin American and Caribbean Health Sciences Literature (Virtual health Library, 1982–2022), Medline (PubMed, 1966–2022), and the Cochrane Collaboration’s Controlled Clinical Trials (Cochrane Central Register of Controlled Trials). The following index terms and their synonyms will be used: Alzheimer’s disease; acupuncture; Western medicine. No language or year restrictions are considered for this study. We will use the validated randomized clinical trials filters created by the Cochrane Collaboration for Medline and Embase. The following databases will also be searched to find eligible studies: Scopus, China Biomedical Literature Database, Wanfang Database, China National Knowledge Infrastructure, and Australian Medical Index. We will also search the gray literature for relevant clinical trials and conference abstracts.

### 2.2. Criteria for inclusion

Studies meeting the following inclusion criteria will be included: Subjects with definite diagnosis of AD; Participants in the experimental group received a combination of acupuncture (including electroacupuncture, needling, moxibustion, and acupressure) and western medicine, while those in the control group received either western medicine alone or acupuncture alone. At least 1 clear outcome was included in the study, such as clinical efficacy, Ability of Daily Living, Traditional Chinese Medicine symptom score, sleep disorder improvement score, Mini-Mental State Examination, mood and behavior score and adverse events.

### 2.3. Criteria for exclusion

The following studies were excluded: animal studies, case reports, reviews, experience presentations, or conference articles, articles with no clear outcome measures or incomplete information, or articles involving other types of dementia in addition to AD.

### 2.4. Study selection process

**Figure 1.**
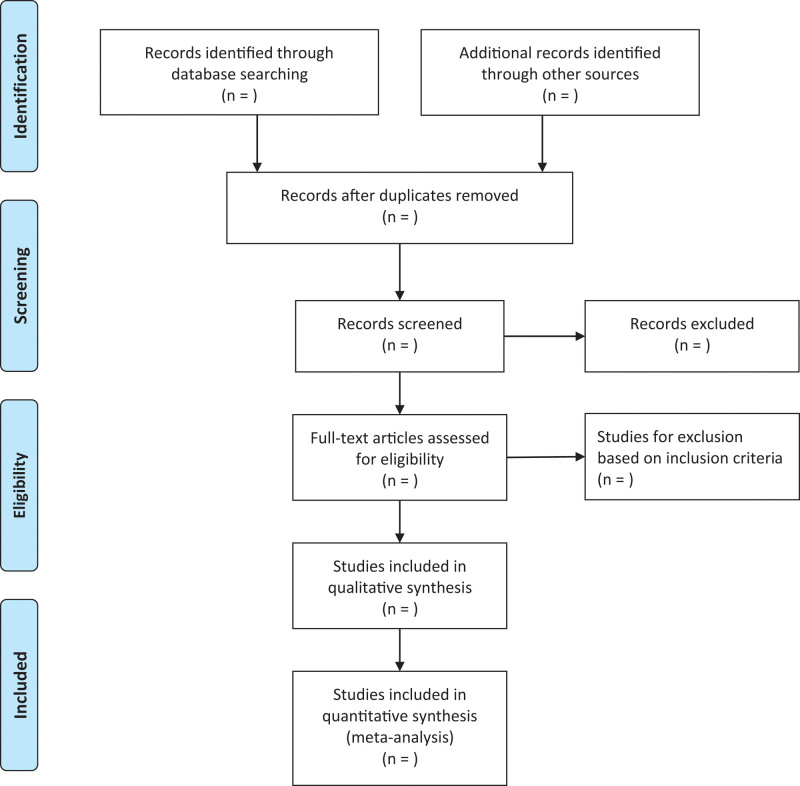
Flow diagram of the studies selection process.

All investigators will receive appropriate training prior to the start of data extraction. Rayyan online literature management software (https://Rayyan.qcri.org) will also be used to screen and manage the literature. After screening a random sample of 50 citations, a Kappa test will be used to calculate interobserver agreement for all studies. If the Kappa value is < 0.75, a second round of training will be performed. Titles and abstracts of identifiable articles will be screened independently by 2 reviewers to exclude reports that clearly do not meet the inclusion criteria. The same reviewers will then independently review the full-text articles to determine eligibility. When disagreements arise, a third author will be asked to evaluate the full text and disagreements will be resolved through group discussion. Figure 1 depicts the study selection process for the preferred reporting items in the flow chart for Systematic Review and Meta-Analysis (PRISMA).

### 2.5. Data extraction

Study data included in our paper will be extracted by 1 reviewer and checked for accuracy by another independent reviewer. The degree of agreement between independent raters will be reported based on the primary outcome, study and population characteristics, kappa coefficients and percentage agreement for risk of bias items. The following descriptive data will be extracted in Excel: country, 1^st^ author, year of publication, intervention and control group, sample size, patient characteristics, primary outcome, secondary outcome, and follow-up time. If there is insufficient information, the authors of the original study will be contacted by phone or email to obtain the missing data if possible. If a study presents incomplete primary outcomes, it will be included in the systematic review only and not in the meta-analysis.

### 2.6. Subgroup and sensitivity analyses

If there may be significant heterogeneity or inconsistency, we will use subgroup and meta-regression analyses to explore possible sources. Subgroup analyses are planned for sample size, age, gender, presence of comorbidities, and type of pharmacological intervention. In addition, we will assess the sensitivity of the primary outcome by analyzing only those studies considered to have a low risk of bias.

### 2.7. Risk of bias

The Cochrane risk of bias tool will be independently used to evaluate the risk of bias of included randomized cohort studies by 2 reviewers. The quality will be assessed by using following 7 items: random sequence generation, allocation concealment, blinding of participants and personnel, blinding of outcome assessment, incomplete outcome data, selective reporting, and other bias. A description of what was reported to have occurred in each study will be provided and the risk of bias for each domain will be judged according to the following 3 categories: “high risk of bias,” “low risk of bias” and “unclear risk of bias “. The potential bias for “sponsor bias” will be assessed as a separate item. As described by Catala-Lopez et al, the overall risk of bias rating for each study will be the lowest rating for any criterion.^[11]^ Two independent review authors will assess the risk of bias in the selected studies. The degree of agreement between the 2 independent raters will be reported. Any disagreements will be resolved through discussion and consultation with the principal investigator.

### 2.8. Data synthesis

We will perform a network meta-analysis on each outcome using Stata Statistical Software V.16 (StataCorp LLC) to compare multiple interventions in a single model simultaneously. We will prioritize pooling direct evidence; however, in the absence of direct comparisons, effect estimates will be provided through indirect comparisons. Given the expected inter-study heterogeneity, we will use a random effects model for each intervention comparison. We will pool the data for each outcome separately using a Bayesian random effects model. For dichotomous outcomes, effect estimates will be calculated using odds ratios with 95% confidence intervals. Continuous outcomes will be expressed as means and standard deviations for each study.

For direct evidence, we will assess heterogeneity by estimating the magnitude of between-study variance using the empirical distribution estimated by Turner et al and Rhodeset al and by quantifying the percentage of variability due to true differences between studies using the I^2^ statistic. We will interpret I^2^ according to the thresholds set by the Cochrane Collaboration Network, and use it as a criterion for combining or not combining results and for additional subgroup analyses.

## 3. Discussion

This systematic review and network meta-analysis will be the 1^st^ to summarize and compare the benefits of different treatment options for patients with AD, using both direct and indirect evidence. This study will show which therapeutic intervention may be the most promising in the management of AD patients. Heterogeneity of treatment interventions across studies, such as drug dose, treatment duration, and acupuncture site, will limit the validity of the results. Nevertheless, we believe that this network meta-analysis will contribute to methodological advances in the field of systematic reviews, as it will be the first study to compare the direct and indirect effects of different Western and Chinese treatment approaches in the management of AD. The results of this network meta-analysis will influence evidence-based treatment decisions, as it will be the basis for recommendations in the management of patients with AD.

## Author contributions

**Conceptualization:** Liumei Yuan.

**Data curation:** Xinran Wei, Yan Tan.

**Fund:** Wei Zhang.

**Investigation:** Chao Ke, Yang Cao, Zhengrong Xie.

**Methodology:** Jiang Pan.

**Supervision:** Wei Zhang.

**Writing – original draft:** Xinran Wei, Yan Tan.

**Writing – review & editing:** Chao Ke.
